# Compositional Analysis of the Dental Biomimetic Hybrid Nanomaterials Based on Bioinspired Nonstoichiometric Hydroxyapatite with Small Deviations in the Carbonate Incorporation

**DOI:** 10.3390/nano12244453

**Published:** 2022-12-15

**Authors:** Pavel Seredin, Dmitry Goloshchapov, Nikita Buylov, Vladimir Kashkarov, Anna Emelyanova, Konstantin Eremeev, Yuri Ippolitov

**Affiliations:** 1 Solid State Physics and Nanostructures Department, Voronezh State University, Universitetskaya Pl. 1, 394018 Voronezh, Russia; 2 Scientific and Educational Center, Nanomaterials and Nanotechnologies, Ural Federal University, Lenin Ave 51, 620002 Yekaterinburg, Russia; 3 Department of Pediatric Dentistry with Orthodontia, Voronezh State Medical University, Studentcheskaya Ul. 11, 394006 Voronezh, Russia

**Keywords:** non-stoichiometric nanocrystalline hydroxyapatite, biomimetic hybrid nanomaterials, carbonate anion, multimode Raman profile model

## Abstract

In our paper, we discuss the results of a comprehensive structural-spectroscopic and microscopic analysis of non-stoichiometric nanocrystalline hydroxyapatite (CHAp) with low carbonate anion content and biomimetic hybrid nanomaterials produced on its basis. It was shown that hydroxyapatite nanocrystals synthesized by chemical precipitation and biogenic calcium source mimic the properties of biogenic apatite and also have a morphological organization of “core–shell” type. The “core” of the CHAp nanocrystal is characterized by an overabundance of calcium Ca/P~1.9. Thus “a shell” with thickness of ~3–5 nm is formed from intermediate apatite-like phases where the most probable are octocalcium phosphate, dicalcium phosphate dihydrate and tricalcium phosphate. The multimode model of the Raman profile of samples CHAp and biomimetic composites for spectral region 900–1100 cm^−1^ proposed in our work has allowed to allocate precise contribution of B-type carbonate substitution, taking into account the presence on a surface of “core” HAp nanocrystal of various third-party intermediate apatite-like phases. The calibration function constructed on the basis of the described model makes it possible to reliably determine small concentrations of carbonate in the structure of hydroxyapatite with the application of Raman express method of diagnostics. The results of our work can inspire researchers to study the processes of induced biomineralization in mineralized tissues of the human body, using non-destructive methods of control with simultaneous analysis of chemical bonding, as well as determining the role of impurity atoms in the functions exhibited by biotissue.

## 1. Introduction

Biological non-stoichiometric nanocrystalline calcium hydroxyapatite (HAp), composing the mineral phases of bone and teeth [1,2], differs dramatically from stoichiometric hydroxyapatite Ca^I^_4_Ca^II^_6_(PO_4_)_6_(OH)_2_ [3]. Thus the properties of mineralized tissue directly depend on the local chemical and atomic composition, stoichiometry, and also defective structure HAp, that is caused by replacement of the basic functional molecular groups/ions in a crystal lattice of bioapatite [2]. Calcium atoms in the crystal lattice of stoichiometric Hap are present in two different coordination positions and they are usually designated as Ca^I^_4_ и Ca^II^_6_, in a dependence on their position and bound with anion complexes of PO_4_ or ОН goups [3]. Presence of impurity atoms results in distortions, imbalance, vacancies and thus, in the general case, structural formula is supplemented with a great number of the ions located in the corresponding positions of a material Ca^I^_4−x_Me^I^_x_Ca^II^_6−y_Me_y_(PO_4_)_6−k_(XO_3_)_k_(OH)_2−m_(Y)_m_ [3,4].

Defective carbonate-substituted hydroxyapatite (cHAp), can be approximately described by the following formula: Ca^I^_4−x_Me^I^_x_Ca^II^_6−y_Me_y_(HPO_4_)_z_(PO_4_)_6−z−k_(CO_3_)_k_(OH)_2−l−m_(Cl)_l_(F)_m_ [4,5,6]. Thus, in biogenic materials Me^I^_x_ are sites in lattice HAp, which can be occupied by atoms of metals Mg, Na, Fe, Sr, etc. Due to imbalance in the cation sub-lattice, the anion sub-lattice also involves substitutions of the following type: PO_4_ group is substituted by HPO_4_ and/or by CO_3_ one, while OH group is substituted by Cl and/or F [4,5,6]. Note that content of the impurity components is varied within 0.02–5%. Such low-concentration substitutions provide bioapatite realization with various biological functions in the mineralized tissue [7], influence mechanical properties, solubility, crystallinity, microstructure, etc. [3,8]. As a result, it allows to adapt the properties of the created functional biomaterials [9], and can also influence the mechanisms of cell formation and resorption [10].

In this regard, the actual task, as well as the subject of modern research is to determine the type of impurity elements in the structure of bioapatite, as well as their concentration and influence on the manifested mineralized tissue properties [3,4,7].

It is well known that the mechanical properties of mineralized tissue are often interpreted by the ratio of apatite to collagen, while tissue dynamics (i.e., maturation and exchange/remodeling) are often interpreted by the ratio of carbonate to phosphate [11]. Thus, replacement of phosphate group PO_4_^3−^ by carbonate-anion CO_3_^2−^ in a crystal lattice of hydroxyapatite essentially influences the observed physical properties, the size of crystallites and the crystallinity of the mineralized tissue [11,12,13]. 

However, simultaneous multi-ionic substitutions in the apatite lattice can often lead both to a synergetic effect and have an opposite effect on a number of properties due to competition between the substituting ions/groups [5,7]. Thus, the works devoted to the study of synthetic materials based on HAp have shown that most of the characteristics (strength, ductility, solubility, etc.) can be controlled by the introduction of impurity atoms of a certain subgroup into the HAp lattice at a given concentration [5,7,9]. With the use of monatomic impurity there is no possibility to attain the direct properties of biogenic apatite which has multiple impurity atoms in its composition [4,5].

On the other hand, an excess of impurity elements leads to a global change in the physical characteristics of apatite and has negative consequences. It is known that the formation of carbonate-substituted forms of hydroxyapatite with high CO_3_^2−^ content leads to increased solubility of tooth enamel, resulting in tooth decay [14,15]. In addition, the excess of fluorine atoms leads, on the contrary, to greater stability and durability, but in its turn can interfere a diffusion of minerals and ions to the surface of teeth that eventually leads to chipping of the dental tissue [5,16]. Therefore, an accurate calculation of the small value (degree) of substitution in the crystal lattice of the synthesized bioapatite can be a convenient tool for predicting the physicochemical and functional properties of substituted hydroxyapatite.

As for the methods related to a precise determination of concentration of the elements in the samples of biological nature one of the most attractive technique for the clinical laboratory seems to be an inductively coupled plasma mass spectrometry (ICP-MS) [17]. In order to determine defects and a degree of substitution in the HAp crystal lattice, including surface-modified HAp, X-ray diffraction [8,18], transmission electron microscopy [19,20], photo-emission synchrotron spectroscopy [21], photoluminescence [22] and electron paramagnetic resonance (EPR) spectroscopy [23] are often employed.

At the same time, there is a large number of works devoted to the express monitoring of the changes in the molecular composition of HAp with different contents of foreign ions [24], employing methods of molecular spectroscopy. For example, the content of carbonate-ion can be determined using the methods of Infrared and Raman spectroscopy [25,26]. In some cases, due to the high sensitivity of Raman spectroscopy and the peculiarities of this technique, carbonate-ion content can be determined with a high accuracy. So, in their work, Awonusi et al. [27] show the use of this method for the analysis of artificial and synthetic apatites. It was established (Spizzirri et al. [28]) that the possible detectable limit of CO_3_ substitution was in the range of 2–7% [28], and in Awonusi et al. it was in the range of 0.3–10.8 wt% [25].

The main problem in the study of natural mineralized tissue and biocomposites is the complicated spectral profile [29]. Overlapping of PO_4_^3−^, CO_3_ bands and their superposition at the spectral contribution of the organic component, as well as a precise determination of the baseline, whose shape will be influenced by the background fluorescence of the sample of biological nature, is the problem of creating the express control techniques [11]. Using mathematical processing and deconvolution of spectral data, it is possible to obtain good convergence in determining CO_3_ content by direct methods of measuring of its concentration [25,28]. However, in the case of small concentrations of ~0.1–2%, there is a need to refine the results using local methods of analysis sensitive to the atomic environment in the crystal lattice, since the spectral characteristics of biogenic samples often change nonlinearly [12,25,29].

A review of the literature in this area shows a small number of papers that investigate the low percentage of substitution in the apatite structure of both biogenic tissues and biocomposites with simultaneous analysis of chemical bonding using vibrational and X-ray microspectroscopy.

Therefore, the purpose of this work was to discuss the results of a comprehensive structural-spectroscopic and microscopic analysis of non-stoichiometric nanocrystalline hydroxyapatite with low carbonate anion content and biomimetic hybrid nanomaterials created on its basis, as well as the spectroscopic express method for determining the low carbonate content in the structure.

## 2. Materials and Methods

### 2.1. Obtaining of the Samples

In our work, we investigated samples of non-stoichiometric nanocrystalline hydroxyapatite (CHAp), as well as biomimetic hydroxyapatite nanocomposites (BHN) based on them, developed for restoration of the enamel and dentin of human teeth. CHAp samples were obtained by chemical precipitation, titrating a concentrated calcium hydroxide (Ca(OH)_2_) solution with 0.3 M orthophosphoric acid (H_3_PO_4_) solution in the atmosphere using our methodology, which employed a biogenic calcium source [20]. Changing the concentration of H_3_PO_4_ in the solution allowed to perform synthesis of nanocrystalline materials with different degrees of non-stoichiometry. Changes in the Ca/P ratio resulted in materials with a calculated CO_3_ content in the nano-CHAp structure within 1.7% < x < 2.0%.

BHN biomimetic composites simulating enamel and dentin properties were prepared using carbonate-substituted hydroxyapatite with a percentage of CO_3_~1.8%. To reproduce the amino acid matrix of enamel and dentin, a set of basic polar amino acids characteristic of the dental matrix (L-arginine hydrochloride, L-histidine, and L-lysinehydrochloride) was used [30,31,32]. The ratio of the mineral component in the biomimetic hydroxyapatite nanocomposites were chosen taking into account the content of apatite in tooth enamel and dentin: ~95 for the BHN-1 composite and ~75% for the BHN-2 composite.

Ten samples of each type were prepared. The characteristics of the samples are shown in Table 1.

### 2.2. Research Methods

High-resolution microscopic studies of non-stoichiometric hydroxyapatite samples were performed using a Libra 120 transmission electron microscope (Carl Zeiss, Aalen, Germany).

Elemental analysis of surface layers of the samples was performed using X-ray photoelectron spectroscopy (XPS.) Experimental XPS spectra were obtained using a Thermo Fisher Scientific K-Alpha XPS spectrometer with a hemispherical analyzer having a 180° dual focus and 128-channel detector. The X-ray source was monochromatized AlK_α_ radiation with a variable beam diameter (50–400 μm) up to 72 W power and charge-compensation capability.

High-resolution diffractometric studies were performed at the ANKA-PDIFF channel of ANKA synchrotron (Karlsruhe, Germany). The radiation source was a rotating magnet generating a magnetic field with an induction of 1.5 T (Ec = 6 keV). Monochromatic radiation corresponding to Cu Ka1 = 1.5405 Å was used in the experiment. The flux per sample of the focused radiation with 10 keV energy was ~2 × 10^16^ W/m^2^ at a current in the channel of 100 mA. The size of the cross section of the beam incident on the sample was ~0.5 × 0.5 mm. X-ray phase analysis (XRD) was performed using the JCPDS-ICDD database.

Raman spectra of the samples were obtained using the RamMix EnSpectr M532 Scientific Edition Raman microscope coupled to an Olympus CX-41 microscope. The study was performed using 532 nm excitation radiation. The power per focused beam surface was 30 mW. Exposure time was of 1 s, the number of averages was 30.

Processing of XRD, XPS, and Raman spectral data, baseline correction, averaging, determination of peaks positions, integral area values, and decomposition into components was performed using Origin software. Statistical analysis of the results was performed using the professional software package SPSS ver. 19 for Windows, SPSS Inc., Chicago, IL, USA. Descriptive statistics in the groups were performed using standard *t*-test and given as mean ± standard deviation.

Preliminary studies have shown that the characteristics (morphological, structural and spectral) for samples of a particular type (in a particular sample) slightly differ from each other. Given this fact in our work, we present sample-specific results: typical microscopic images, as well as sample-averaged diffractograms and spectra.

## 3. Results and Discussion

**Figure 1:**
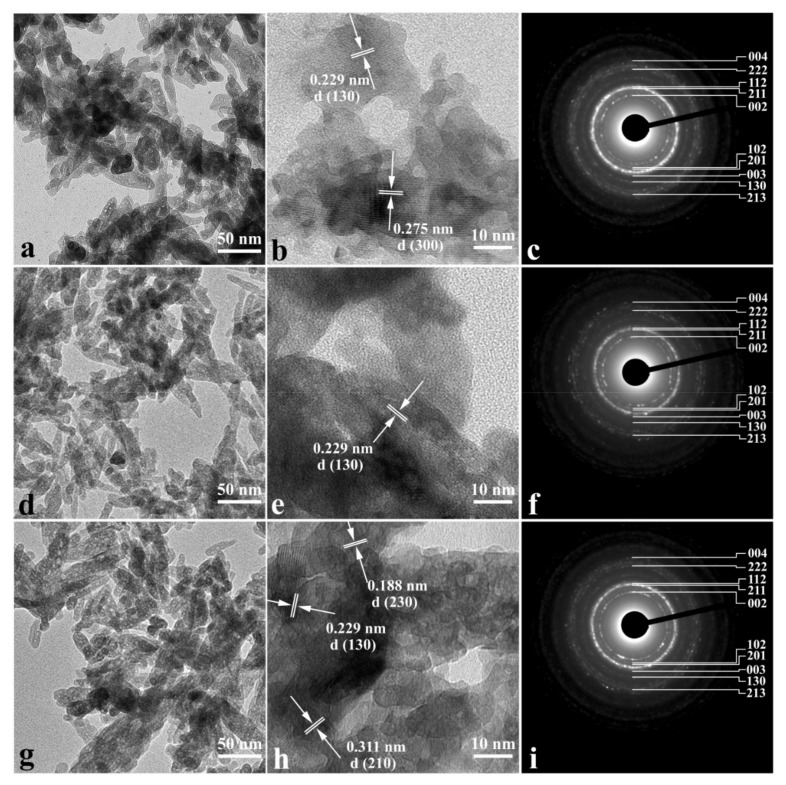
Results of high-resolution transmission electron microscopy with different magnifications (**a**,**b**,**d**,**e**,**g**,**h**) and SAED patterns (**c**,**f**,**i**) of non-stoichiometric nanocrystalline hydroxyapatite samples. (**a**–**c**)—CHAp-1; (**d**–**f**)—CHAp-2; (**g**–**i**)—CHAp-3.

The results of high-resolution transmission electron microscopy (HRTEM) presented in Figure 1 confirmed that the CHAp samples synthesized at three different Ca/P ratios consist of needle-shaped nanoparticles of similar size (20 × 50 nm), mimicking those of biogenic apatite [33].

Figure 1b,e,h shows HRTEM images of agglomerates consisting of thin elongated crystals, where the atomic planes that correspond to the interplanar distances characteristic of (130), (300), (230) and (210) hydroxyapatite planes are clearly visible [34,35]. Each of these planes is perpendicular to the [0001] direction of the HAp crystal lattice, which, as seen in HRTEM images (Figure 1b,e,h), is the preferred growth direction of HAp nanocrystals. The selected area electron diffraction (SAED) results presented in Figure 1c,f,i indicate at the different crystallinity in HAp needle-shaped nanoparticles [34,35] obtained at three Ca/P ratios. This is evidenced by the diffraction rings characteristic of the HAp nanocrystalline shape (Figure 1c,f,i) [35,36]. The results of HRTEM studies confirm the morphological similarity of the nanocrystalline hydroxyapaptite that we synthesized with human tooth enamel and dentin apatite [33].

**Figure 2:**
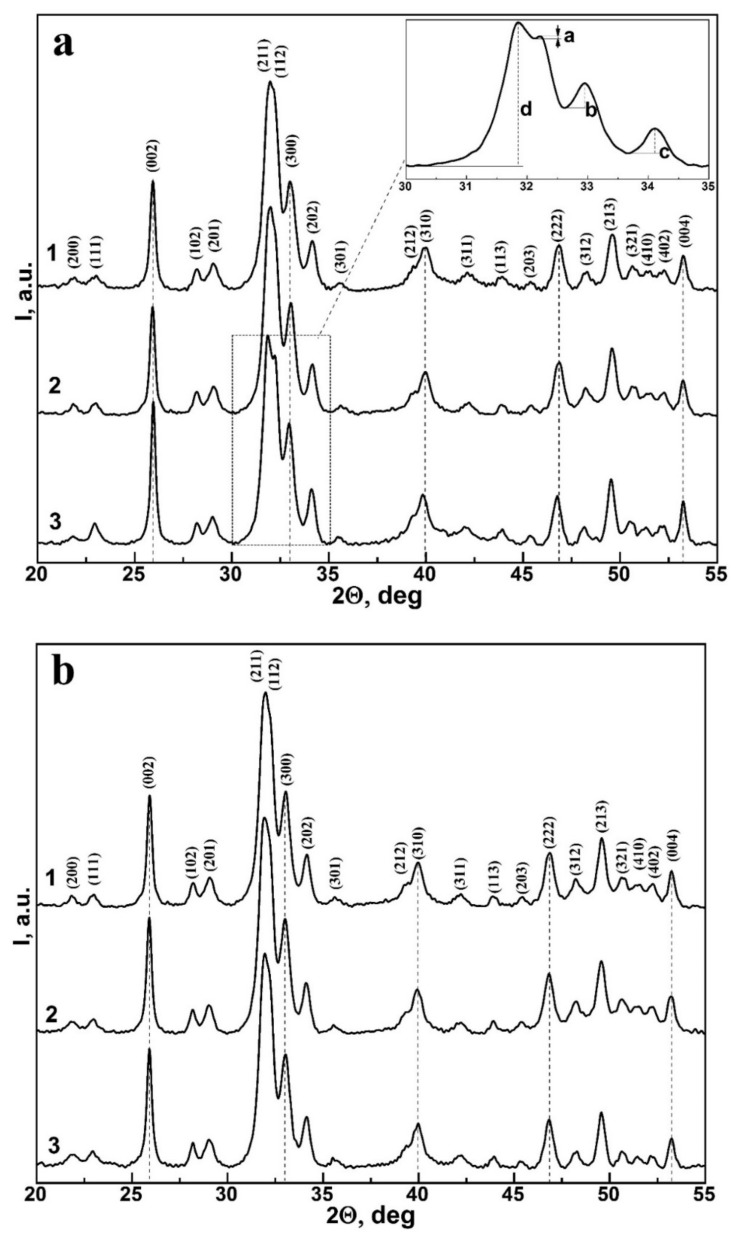
X-ray diffraction (XRD) pattern of (**a**) 1—CHAp-1, 2—CHAp-2, 3—CHAp-3 (**b**) 1—CHAp-2, 2—BHN-1, 3—BHN-2.

The phase composition of the CHAp samples was also confirmed using X-ray diffraction. Figure 2a shows the diffractograms of non-stoichiometric hydroxyapatite samples at different Ca/P ratios, as well as of biomimetic composites (Figure 2b). Crystallographic identification showed that the diffraction pattern of the studied materials presents the same set of characteristic peaks of high intensity, corresponding to only one crystalline phase—calcium hydroxyapatite. In addition, it is well seen that there is almost no noticeable difference in the X-ray diffraction pattern of non-stoichiometric hydroxyapatite samples and biomimetic composites.

The same set of broad reflexes is observed at the diffractograms, which means the small size of crystallites [33]. However, it is clearly seen that with an increase in the Ca/P ratio the doublet of (211) and (112), reflexes begin to be resolved at the diffractograms and the relative intensity of reflexes (211), (122), (300) and (202), and their width change as well. This, in turn, reveals a change in the size of HAp crystallites, as well as the crystallinity value of the materials [35,37]. The crystallinity index, which was proposed by Person et al. [38], refers to the fraction of the crystalline phase present in the bulk of a sample under study. The crystallinity index (CI) for hydroxyapatite crystals can be calculated using the height of the diffraction peaks a, b, c and d for reflexes (112), (300), (202) and (211), respectively [39]. The main problem of the proposed technique is the correct separation of these peaks in the diffraction picture. In this case, the height of each maximum is measured as shown in the Tab to Figure 2a.

Thus, the CI crystallinity index can be determined from the following equation:

(1) $$x_{c}=\frac{a+b+c}{d}100\%$$

Calculation of the index of crystallinity (see Table 2) shows that for the samples of non-stoichiometric hydroxyapatite CHAp this value lies in the range of 27 ± 1.11%–36% ± 1.48% and increases with a of decreasing Ca/P ratio. This, in turn confirms the fact that we obtained nanosized HAp crystals.

Note that the crystallinity value of the samples of BHN biomimetic composites coincides with the value characteristic of CHAp-2 nanocrystalline apatite powder used for the synthesis of BHN-1 and BHN-2 samples simulating the properties of enamel and dentin.

**Figure 3:**
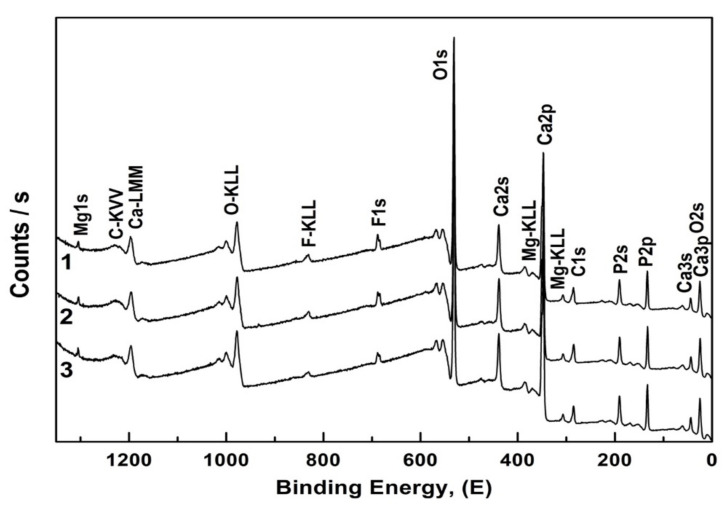
Overview XPS spectra of non-stoichiometric hydroxyapatite samples. 1—CHAp-1; 2—CHAp-2; 3—CHAp-3.

Detailed information about the chemical state of the elements in the samples was obtained using X-ray photoelectron spectroscopy (XPS). Figure 3 shows overview XPS spectra for nanocrystalline hydroxyapatite CHAp samples with three different Ca/P ratios. It should be noted that in our work we do not give XPS spectra of biomimetic materials, because we were not able to obtain a qualitative result.

The analysis showed that XPS spectra were dominated by the narrow emission peaks, which were identified according to the core state of surface atoms, and detected the presence of the following elements in all CHAp samples: Ca, P, O, C. Mg and F were also detected above the detection limit. In addition, broad less intense lines corresponding to Auger transitions of the detected elements are observed in the spectra.

Note that the XPS spectra reveal both structurally bound carbon, due to its embedding into the crystal lattice with a carbonate anion, and also as an accidental contaminant from the atmosphere. The described spectral features are observed for the samples synthesized using liquid-phase synthesis and/or having introduced impurity atoms [40,41,42,43].

**Figure 4:**
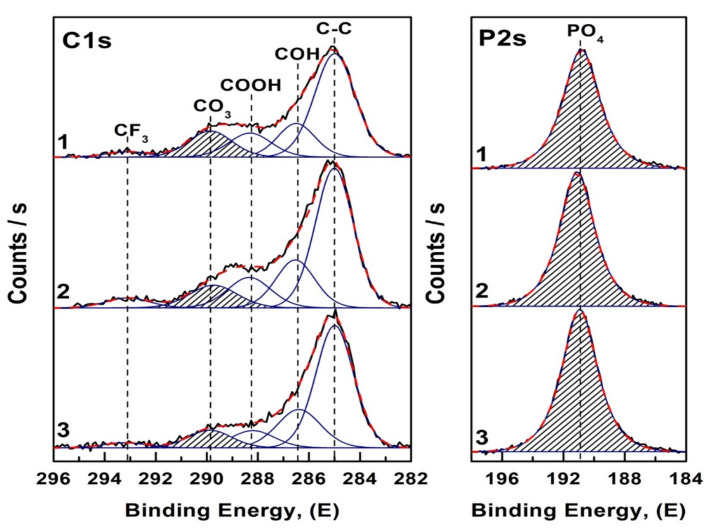
XPS spectra of core level C1s (**left**) and P2s (**right**) of non-stoichiometric hydroxyapatite samples. 1—CHAp-1; 2—CHAp-2; 3—CHAp-3.

Figure 4 shows XPS scans in the C1s and P2s spectral line regions obtained at high resolution from the surface layers of CHAp samples. Spectral data processing (normalization, baseline removal, determination of the binding energy and decomposition of the profiles into components) was performed using Casa XPS software. The spectra were normalized by the C-C bond line position of 285.0 eV.

The analysis of the obtained results showed that the main components in the spectral profile of the C1s line (Figure 4) correspond to the bonds of carbon with oxygen and hydrogen—COH, COOH, and CO_3_ [44,45,46,47]. The maximum in the ~293 eV region belongs to the C-F3 bonds [48,49], and its appearance in the spectra is associated with surface contamination of the samples. At the same time, in the spectral profile of the P2s line (Figure 4) there is only one maximum corresponding to the oxygen environment of phosphorus PO_4_ [50].

It should be noted that according to the experimental data as the value of Ca/P ratio decreases in the spectra of non-stoichiometric hydroxyapatite CHAp samples there is an increase in the integral bond intensity of PO_4_ (Figure 4, right) and simultaneously a decrease in the integral bond intensity of CO_3_ (Figure 4, left).

Using the XPS data based on the ratio of integral intensities of Ca2p_3/2_ and P2p_3/2_ lines, taking into account the factors of relative element sensitivity, a semi-quantitative analysis was performed and the Ca/P ratio in CHAp samples was estimated. 

For this purpose, the known relation [51] was used:

(2) $$\frac{Ca}{P}=\frac{I_{Ca}}{I_{P}}\;\frac{\sigma_{P}}{\sigma_{Ca}}$$

where *I_Ca_* and *I_P_* are the integral intensities of the XPS lines Ca2p_3/2_ and P2p_3/2_ in the spectra, *σ_Ca_* and *σ_P_* are relative sensitivity factors for the 2p_3/2_ calcium and 2p_3/2_ phosphorus levels (*σ_Ca_* = 3.350, *σ_P_* = 0.789).

The integral areas of the Ca2p_3/2_ and P2p_3/2_ line maxima were determined after Shirley background correction [52] in the range of the binding energies of the peaks of interest.

According to the results of evaluation of the Ca/P ratio near the surface of all the investigated samples supersaturated content of phosphorus Ca/P~1.3–1.4 is observed. At the same time as it is known stoichiometric Ca/P ratio for HAp is ~1.67 [53]. This result is associated with the peculiarities of the methodology used by us to obtain nanocrystalline non-stoichiometric hydroxyapatite [20]. As it was described earlier (see Section “Materials and Methods. Obtaining samples”) calcium alkali Са(ОН)_2_ synthesized from a biogenic source was titrated with a constant rate by a weak solution of phosphoric acid for a long time. Thus, formation of hydroxyapatite nanocrystals proceeds due to formation of Posner’s clusters of Са_9_(РО_4_)_6_ [54]. However, the final Ca/P ratio depends on the ionic composition of the solution and may vary due to excessive calcium content in it, as well as the inclusion of foreign ions replacing calcium and phosphate groups into the Posner’s clusters [54]. In our case, formation of nanocrystals occurs at initially high content of calcium in the solution and lack of PO_4_ groups that can be compensated by inclusion of CO_3_ anion in structure of newly formed hydroxyapatite. As a result, the “core” of the CHAp nanocrystal may be overabundant in calcium, resulting in a high Ca/P ratio of ~1.9. As the H_3_PO_4_ content in the mixture increases, the solution as a whole is neutralized by the formation of hydroxyapatite of a given non-stoichiometry. 

It should be noted as that it has been previously reported, biomimetic CHAp nanoparticles may represent a crystalline apatite “core” coated with an ACP/OCP-like layer [55], that is actually correlated with our data obtained previously. Using Raman spectromicroscopy and synchrotron XANES spectroscopy, we have already shown that on the surface of the obtained bioinspired hydroxyapatite nanocrystals, calcium atom is more typical of being in the position associated with phosphorus-oxygen tetrahedrons [32]. Therefore, in the surface layers (“shell”) of the formed nanocrystals of ~5 nm lower Ca/P ratio can be observed. 

To verify this fact, XPS studies were performed on the CHAp-2 sample after two-step etching of the sample surface with argon ions at an energy of 4 keV for 1 s. The choice of this sample for the analysis was due to the fact that it was used for the synthesis of biomimetic composites.

**Figure 5:**
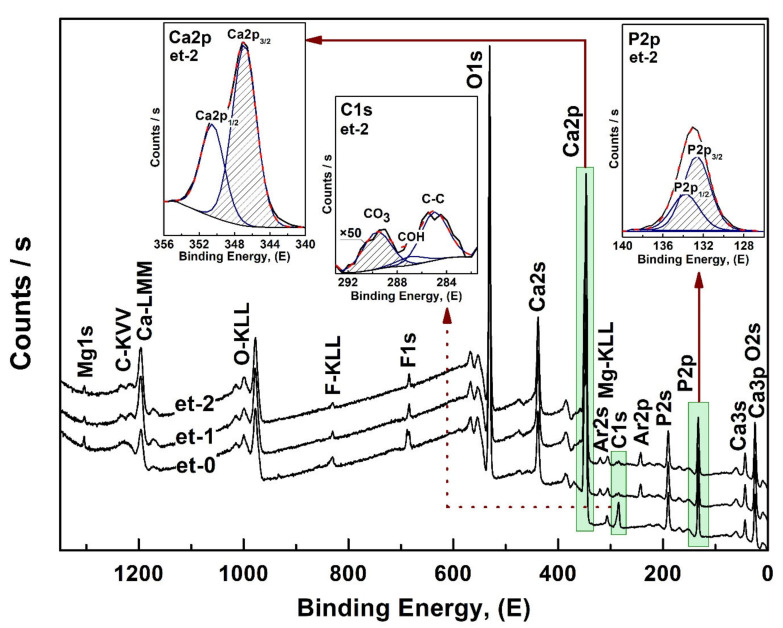
Overview XPS spectra of the CHAp-2 sample before and after its etching. et-0—spectrum before etching; et-1—spectrum after the first etching step; et-2—spectrum after the second etching step. The tabs show the XPS decompositions of the Ca2p_3/2_ and P2p_3/2_ lines into the components for the spectrum of et-2 (after the second etching step).

The overview XPS spectra after the etching procedure are shown in Figure 5. It can be clearly seen that after etching (scans et-1 and et-2, Figure 5) the elemental composition of the samples was not changed, and all the previously detected maxima are present in the spectra (et-0, Figure 5). At the same time, as a consequence of the etching procedure, the Ar 2s and Ar 2p lines at binding energies of 320 eV and 245 eV, respectively, are present in the XPS overview spectra (see Figure 5). In addition, the intensity of the carbon lines associated with the surface contamination of the samples decreases rather markedly. However, the most important fact here is that the intensity of the phosphorus P2p_3/2_ line did not change with etching, while the intensity of the calcium Ca2p_3/2_ line increased significantly.

Calculation of the Ca/P ratio based on (2) showed that after two etch stages this value is at Ca/P~1.85, which in turn coincides with the calculated similar value for this sample (see Table 1). It should be noted that the depth of the XPS analysis is of ~3 nm, and the effective etch depth is of ~2–3 nm.

It is well known that replacement of PO_4_^3−^ and OH-groups by CO_3_^2−^ anion separately or simultaneously and in the different proportions causes deviation of Ca/P ratio [53]. Thus, changes occurring in the chemical composition of mineral apatite-like phases, taking into account hierarchical features of their structure, can be easily and quickly detected with application of Raman microspectroscopy.

**Figure 6:**
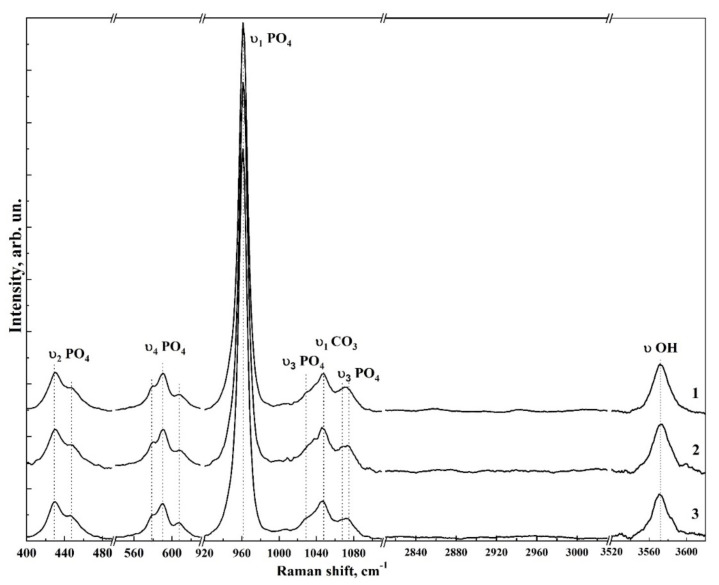
Overview Raman spectra for non-stoichiometric hydroxyapatite samples. 1—CHAp-1; 2—CHAp-2; 3—CHAp-3.

**Figure 7:**
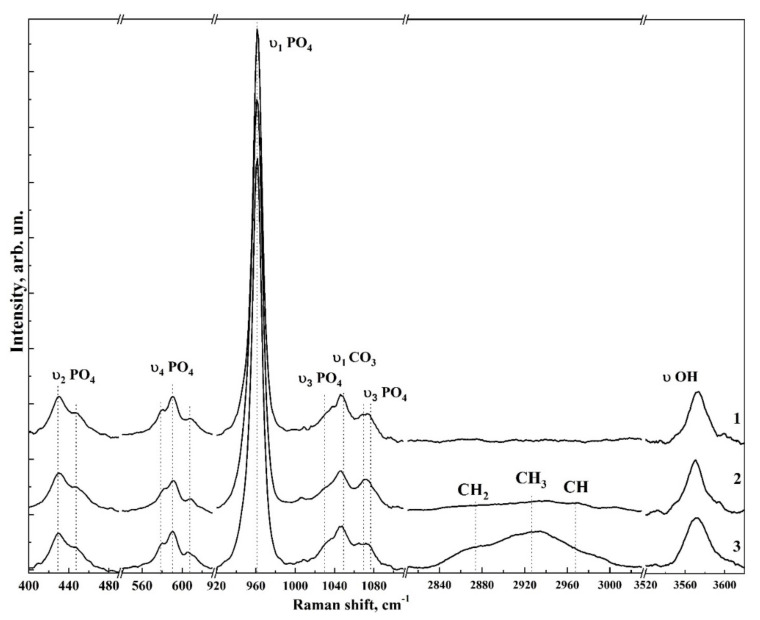
Overview Raman spectra of biomimetic composite samples simulating the properties of enamel and dentin as well as the CHAp-2 sample used for their synthesis. 1—CHAp-2; 2—BHN-1, 3—BHN-2.

Figure 6 and Figure 7 show typical Raman spectra of nanocrystalline non-stoichiometric hydroxyapatite CHAp samples with different Ca/P ratios and spectra of biomimetic composites. The Raman spectra of all our samples had the same kind of the luminescence background typical for HAp samples [56]. Spectra in the Figure 6 and Figure 7 are presented after correction of the baseline with the use of polynomial approximation performed in the Origin program suite. The spectra are presented in the bands 400–630, 920–1100 cm^−1^ and 2820–3620 cm^−1^ where the main vibrations related to mineral and organic components of the samples are located. The most intense band in the spectrum (960 cm^−1^) was normalized beforehand.

Analysis of Raman spectra shows that the most intense peaks in the spectrum belong to the characteristic vibrations of the phosphate ion PO_4_^3−^ in the lattice of hydroxyapatite. Thus, the mode in the region of 960 cm^−1^ is attributed to the valence vibration υ_1_ of PO_4_^3−^ [29,57,58,59,60,61]. In addition, υ_2_ and υ_4_ PO_4_ bending modes are active in Raman spectra, the first of which appears as a clearly distinguishable doublet at ∼430 cm^−1^ and 448 cm^−1^, and the second as a superposition of four maxima at ~580 cm^−1^, 590 cm^−1^, 608 cm^−1^ and 619 cm^−1^. The peaks between 1000 and 1100 cm^−1^ belong to PO_4_ υ_3_ stretching mode vibrations.

Observed fine structural effects (broadening of the band in Raman spectra, band splitting and energy shifts) in nanomaterials [62], including calcium hydroxyapatite are known to correlate rather often with the size of crystallites [63]. We did not observe the effects of broadening of Raman bands due to the changes in crystallite sizes, since in our synthesized samples their values are very similar. Taking this fact into account the fine lattice properties in Raman spectra (broadening of the bands and their shift) are mainly connected with inclusion of the foreign ions, particularly, with carbonate anion [28].

The increased content of carbonate groups in the structure of hydroxyapatite is known to cause not only the broadening of all bands in its Raman spectrum, but also leads to the appearance of additional modes. So inclusion of СO_3_^2−^ in the crystal lattice of HAp, occurring during PO_4_^3−^ group substitution (B-type substitution, typical for biogenic materials [25,28,57,60,64,65]) leads to the appearance of maximum at about 1068–1070 cm^−1^ (Figure 7). At the same time, inclusion of СO_3_^2−^ in the HAp lattice instead of OH group (A-type substitution, characteristic of natural enamel/dentin [28,57,59]), leads to the appearance of a band in the region of 1101–1104 cm^−1^, which is not observed in our experimental spectra (Figure 7). This once again confirms the fact that nanocrystalline HAp with B-type substitution can be obtained using our synthesis technique [20,31].

Combination of the scattering spectra also provides information about hydroxyl bonds in the apatite lattice which appear in the Raman spectra as a stretching mode around ∼3570 cm^−1^ (see Figure 6 and Figure 7) [66].

As for the contribution from the organic component, in the spectra of biomimetic composites (see Figure 7) in the region of 2840–3000 cm^−1^ C-H vibrations are clearly visible, and their intensity depends on the amino acid matrix content in the biocomposite. 

The frequencies of the main modes in the Raman spectra as well as their assignment to the molecular groups and ions of mineral and organic components of the synthesized materials and biomimetic composites are presented in Table 3. The comparison was made on the basis of known literature data [25,28,29,59,64,65,67,68,69,70].

In the Raman spectrum of hydroxyapatite the position of the phosphate bands is quite well established, while the correlation of the carbonate bands is not consistent [10,25,57]. The reason for this may be not only due to the changes related with the content of carbonate groups, but also with the phonon set characteristic of the accompanying phosphate phases (apatite-like surroundings) in the spectra, as well as their influence on the presence of carbonate in the HAp lattice [43,68,70,73,80].

Therefore, we decomposed the Raman spectral profile in the region of 900–1100 cm^−1^ into components to determine the fine structural properties. Decomposition of the experimental spectral curves was carried out, taking into account the known data on the mode composition in this part of the spectrum (See Table 3). Individual modes were simulated using the Pearson7 function, which best fits the shape of Raman lines. Extremes were determined using second and fourth derivatives as well as taking into account theoretical calculations [29,81] and experimental works [25,28,59,64,65,67,68,69,70].

In contrast to Awonusi et al. [25], where simulations were performed for the 1020–1100 cm^−1^ region, in our work a wider region of 900–1100 cm^−1^ was used for deconvolution due to the presence of the features correlated with weak phosphates. As it was shown in [28,55], consideration of the features in the broad spectral profile makes it possible to correctly determine the intensity and area of the spectral curves in relation to each other.

Modeling was performed on the basis of the approach tested by us in a number of works [55,82,83], taking into account the limitations imposed on the procedure for determining the number of maxima in the spectrum, calculating and removing the baseline and determining the convergence of the decomposition result. All these features made it possible to find the necessary criterion for the convergence and reproducibility of the simulation results, as well as to ensure the uniqueness of the spectral profile decomposition, which was confirmed by multiple independent simulations for the samples included in one or another group.

The decomposition quality was determined taking into account the error minimization criterion (χ-square) depending on the mode composition of the spectral band (number of maxima) and the range of acceptable parameters (peak height, width, center and shape).

**Figure 8:**
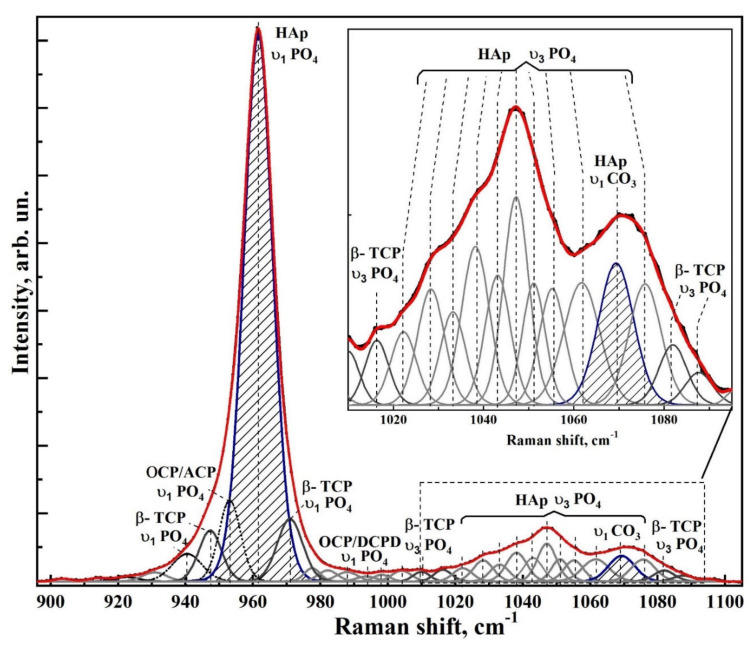
Experimental Raman spectral profile in the region of 900–1100 cm^−1^ and its deconvolution for the sample of non-stoichiometric apatite CHAp-1. The inset shows the spectral region 1010–1095 cm^−1^.

**Figure 9:**
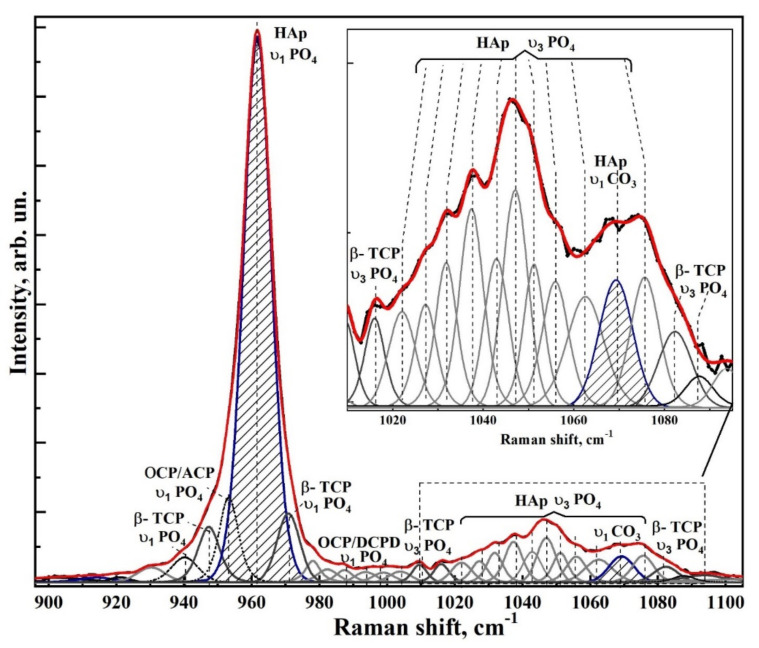
Experimental Raman spectral profile in the region of 900—1100 cm^−1^ and its deconvolution for the sample of non-stoichiometric apatite CHAp-2. The inset shows the spectral region 1010—1095 cm^−1^.

**Figure 10:**
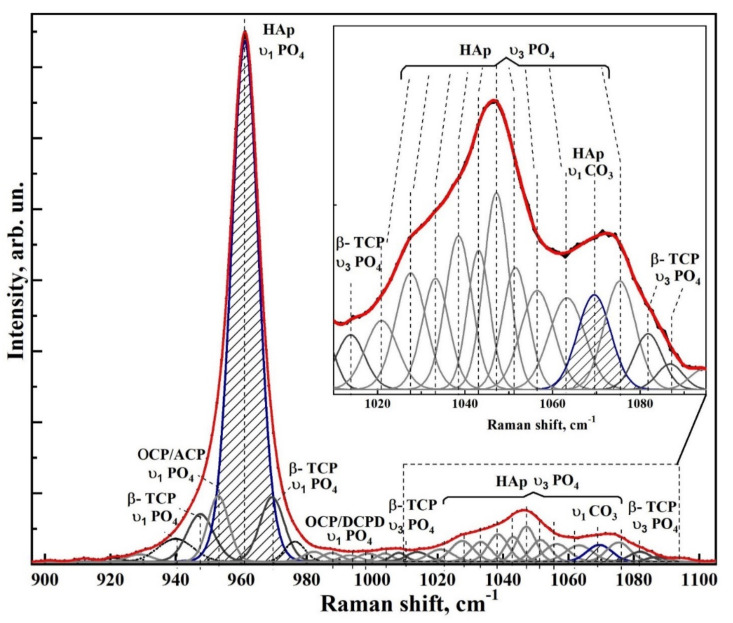
Experimental Raman spectral profile in the region of 900–1100 cm^−1^ and its deconvolution for the sample of non-stoichiometric apatite CHAp-3. The inset shows the spectral region 1010–1095 cm^−1^.

**Figure 11:**
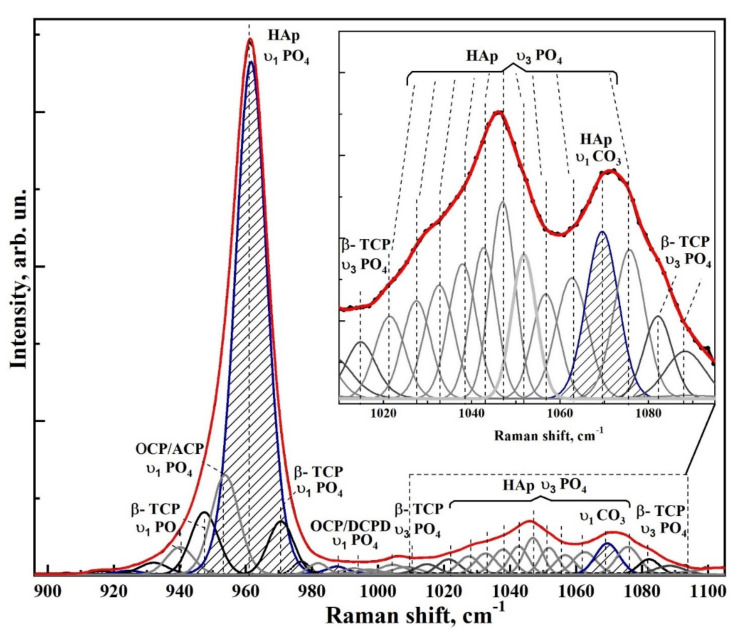
Experimental Raman spectral profile in the region of 900–1100 cm^−1^ and its deconvolution for the biomimetic composite sample BHN-1. The inset shows the spectral region 1010–1095 cm^−1^.

**Figure 12:**
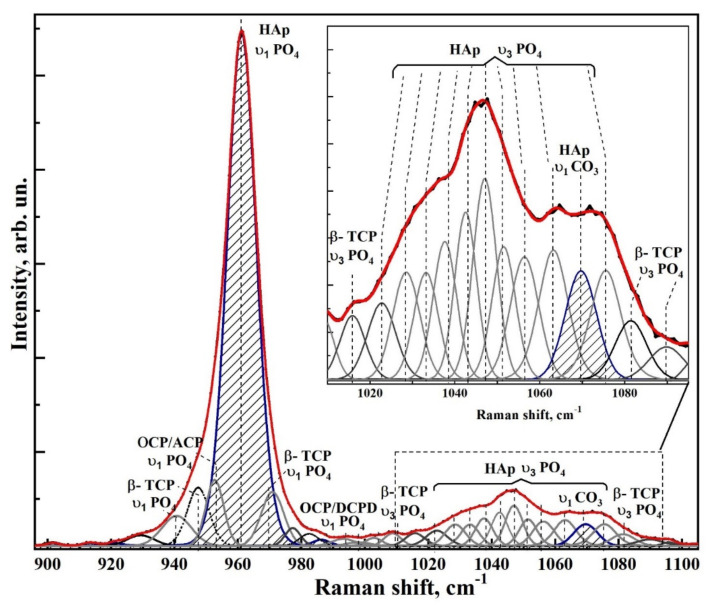
Experimental Raman spectral profile in the region of 900–1100 cm^−1^ and its deconvolution for the biomimetic composite sample BHN-2. The inset shows the spectral region 1010–1095 cm^−1^.

Deconvolution of spectral profiles in the region of 900–1100 cm^−1^ for samples of non-stoichiometric hydroxyapatite, as well as biomimetic composites is shown in Figure 8, Figure 9, Figure 10, Figure 11 and Figure 12. As a result of modeling, not only the modal composition of the spectral region (number of maxima), but also their shape, intensity, width and position are determined. It is clear that our proposed model corresponds well to the width of the spectral profile, as well as it gives a better coincidence with the experimental spectrum.

The results of spectra deconvolution showed that both spectral position and full width at half-height (FWHM) of phosphate modes depend on the sample type. Thus, in the spectra of nanocrystalline hydroxyapatite samples, the FWHM and the frequency of the main phosphate maximum υ_1_ PO_4_^3−^ increase with decreasing Ca/P ratio (see Table 4), which is in agreement with the data from Awonusi et al. [25]. At the same time, for biomimetic composites FWHM of the same mode significantly exceeds the similar value for the sample CHAp-2, used for their creation. This is a consequence of the interaction of HAp with the organic complex of the composite.

As for the spectral region 1010 to 1095 cm^−1^, as presented in Awonusi [25], the model with seven peaks is optimal for describing all of its features. However, according to the results of our simulations, the spectral profile in this region contains a much larger number of components. Among these peaks are not only the maxima correlated with the ν_3_ vibrations of the phosphate ion PO_4_^3−^ hydroxyapatite, as well as the band associated with the carbonate anion CO_3_ (B-type substitution), but also a set of low-intensity modes belonging to the intermediate phosphates (see Table 3). Thus, amorphous calcium phosphate (ACP), octacalcium phosphate (OCP), **ß**-tricalcium phosphate (**ß-TCP**), and Dicalcium phosphate dihydrate (DCPD) are detected in all spectra (see Table 3). The appearance of weak phosphate phases in the composition is associated with the peculiarities of the obtained CHAp samples. In the process of synthesis of non-stoichiometric hydroxyapatite used by us formation of apatite proceeds through formation of intermediate phases [84], among which the most probable are octocalcium phosphate, dicalcium phosphate dihydrate [85] and tricalcium phosphate. Thus, the specified phases during crystallization of hydroxyapatite can appear on the surface (“a shell”) of formed nanocrystals in a small amount—(~5%). This is confirmed on the basis of analysis of the ratios of intensities between the maxima assigned to the intermediate phosphates and mod υ_1_ PO_4_^3−^ HAp of crystal in the spectra (Figure 8, Figure 9, Figure 10, Figure 11 and Figure 12). Taking into account the fact that in the elemental composition of CHAp samples, impurities of foreign ions were found (see Figure 3, XPS data), these atoms can also be a part of the intermediate phases. This in turn leads to a shift in the position of the vibrational modes attributed to the P-O bonds of octocalcium phosphate and tricalcium phosphate, as well as to the broadening of these maxima in the Raman spectra (see Figure 8, Figure 9, Figure 10, Figure 11 and Figure 12) [72]. It should be also noted that the dimensional factor, i.e., the nanocrystalline state of these phases, also contributes to the broadening of the Raman lines. Note that XRD scans of the CHAp samples do not show any reflections from the nanoscale intermediate phases accompanying HAp.

Deconvolution of the spectral profile allowed us not only to clarify the fine structural properties and mineral composition of HAp-based composites, but also to determine the percentage of carbonate anion in the structure of biomimetic nanomaterials.

It is known that the half-width of the phosphate mode ν_1_ correlates with the carbonate content in apatite, i.e., an increased carbonate content leads not only to a decrease in the mineral crystallinity but also to an increase in the FWHM of the phosphate band [86]. However, this model is not sufficiently accurate. Therefore, to determine the percentage of carbonate in hydroxyapatite, we relied on the model proposed by Awonusi et al. [25]. In this work, to calibrate the percentage of carbonate in the sample, we calculated the carbonate–phosphate ratio based on the integral areas of the corresponding maxima in the spectrum $\sim \frac{1071\;{\mathrm{cm}}^{-1}}{960\;{\mathrm{cm}}^{-1}}$.

Awonusi et al. [25] pointed out the linear dependence of the calibration function for the carbonate level in apatite in a fairly wide range (0.1–10%).

In our work, using the results from John-David P. McElderry [12], P.G. Spizzirri et al. [28] and Grunenwald et al. [87], we modified the Awonusi model [25]. A new calibration function with high correlation (R^2^ = 0.97) was constructed for the range of low concentrations of carbonate (0.03–3%), which has an exponential form:

(3) $$\mathrm{Wt}\%\left({\mathrm{CO}}_{3}\right)=0.249e^{5.5x}+1.189e^{5.511x}$$

where x is the ratio of the integral areas of carbonate-phosphate peaks.

Using this function, the carbonate content of our samples was calculated (values are presented in Table 4).

The results obtained on the basis of the new correlation model are in a good agreement with the XPS analysis data for the “core” of HAp nanocrystals, as well as with the calculated values (Table 1).

It should be noted that in HAp nanocrystals the “shell” has a large number of structural defects and has an uncompensated charge. As a consequence, the “shell” should play a weighty role for conjugation with the amino acid matrix in the formation of bionanocomposites. In the biomimetic composites that we studied, the redistribution of components detected in the Raman spectra in the spectral region of 1010–1095 cm^−1^, indicated at the changes just occurring in the “shell” composition, while the “core” remains structurally and compositionally stable. In this case the variation of the carbonate-anion CO_3_ content occurs due to the inclusion of carbon in the structure of the near-surface non-stoichiometric apatite-like phase as the most probable way.

Summarizing the results obtained, we note that our work can be a tool to not only for assessment of the influence of impurity elements on the structure of the matter, to determine the concentration of impurity in a small volume, but also to observe structural changes in bioapatite materials with a high degree of accuracy.

## 4. Conclusions

In our paper we discuss the results of a comprehensive structural-spectroscopic and microscopic analysis of non-stoichiometric nanocrystalline hydroxyapatite (CHAp) with low carbonate anion content and biomimetic hybrid nanomaterials created on its basis.

It was shown that hydroxyapatite nanocrystals synthesized by chemical precipitation and biogenic calcium source mimic the properties of biogenic apatite and also have the morphological organization of “core–shell” type. The “core” of the CHAp nanocrystal is characterized by an overabundance of calcium Ca/P~1.9. Thus, “a shell” with a thickness of ~3–5 nm is formed from intermediate apatite-like phases among which the most probable ones are octocalcium phosphate, dicalcium phosphate dihydrate and tricalcium phosphate.

The proposed multimode model of the Raman profile of samples CHAp and biomimetic composites for the spectral region 900–1100 cm^−1^ proposed in our work has allowed to allocate a precise contribution of B-type carbonate substitution, taking into account presence on a surface of the “core” of the HAp nanocrystal of various third-party intermediate apatite-like phases. The new calibration function with high correlation (R^2^ = 0.97) was constructed on the basis of the described model making it possible to reliably determine small concentrations (0.03–3%) of carbonate in the structure of hydroxyapatite with application of Raman express method of diagnostics.

The results of our work can inspire researchers to study the processes of induced biomineralization of mineralized tissues of the human body using non-destructive methods of control with simultaneous analysis of chemical bonding, as well as determining the role of impurity atoms in the functions of biotissue.

## Figures and Tables

**Table 1:** Description of the tested samples.

Samples	Description	Ca/P (Estimated)
CHAp-1	carbonate substituted hydroxyapatite 1	1.95 ± 0.05
CHAp-2	carbonate substituted hydroxyapatite 2	1.85 ± 0.05
CHAp-3	carbonate substituted hydroxyapatite 3	1.75 ± 0.05
BHN-1	CHAp-2 (~95%) + a set of basic polar amino acids (~5%)	1.85 ± 0.05
BHN-2	CHAp-2 (~75%) + a set of basic polar amino acids (~25%)	1.85 ± 0.05

**Table 2:** CI crystallinity index of non-stoichiometric nanocrystalline hydroxyapatite samples and biomimetic composites.

Sample	CHAp-1	CHAp-2	CHAp-3	BHN-1	BHN-2
$x_{c}$	27.8 ± 1.11%	34.8 ± 1.42%	36.1 ± 1.48%	34.7 ± 1.40%	34.8 ± 1.52%

**Table 3:** Frequencies of the main modes in the Raman spectra and their assignment.

Vibrations	Wavenumber, cm^−1^	Assignment	References
υ_2_ PO_4_^3−^ HAp	431	O-P-O bending, υ_2_	[25,28,29,59,64,65,67,68,69,70]
υ_2_ PO_4_^3−^ HAp	447	O-P-O bending, υ_2_	[25,28,29,59,64,65,67,68,69,70]
υ_4_ PO_4_^3−^ HAp	579	O-P-O bending, υ_4_	[25,28,29,59,64,65,67,68,69,70]
υ_4_ PO_4_^3−^ HAp	590	O-P-O bending, υ_4_	[25,28,29,59,64,65,67,68,69,70]
υ_4_ PO_4_^3−^ HAp	607	O-P-O bending, υ_4_	[25,28,29,59,64,65,67,68,69,70]
υ_4_ PO_4_^3−^ HAp	614	O-P-O bending, υ_4_	[25,28,29,59,64,65,67,68,69,70]
υ_1_ PO_4_^3−^ ß-TCP	938–940	ß-tricalcium phosphate	[71,72]
υ_1_ PO_4_^3−^ ß-TCP	948	ß-tricalcium phosphate	[19,67,71,72,73]
υ_1_ PO_4_^3−^ACP/OCP	953–954	amorphous calcium phosphate/octacalcium phosphate	[55,74,75,76]
υ_1_ PO_4_^3−^ HAp	961–962	P-O stretching	[25,28,29,59,64,65,67,68,69,70]
υ_1_ PO_4_^3−^ ß-TCP	970–971	ß-tricalcium phosphate (ß-TCP)	[19,67,71,72,73]
	977		
	982		
υ_1_ PO_4_^3−^DCPD	985–987	Dicalcium phosphate dihydrate (DCPD)	[57,67,68,77]
υ_1_ PO_4_^3−^ OCPυ_3_ PO_4_^3−^ ß-TCP	1004–1005	octacalcium phosphate (OCP)/Dicalcium phosphate dihydrate (DCPD)/ß-tricalcium phosphate (ß-TCP)	[67,70,72,77]
υ_1_ PO_4_^3−^ OCP	1009–1010	octacalcium phosphate (OCP)	[67,68,77]
υ_3_ PO_4_^3−^ ß-TCP/OCP	1014–1016	octacalcium phosphate (OCP)/ß-tricalcium phosphate (ß-TCP)	[67]
υ_3_ PO_4_^3−^ HAp	1025–1028	P-O antysym stretching	[25,28,29,59,64,65,67,68,69,70]
υ_3_ PO_4_^3−^ HAp	1033	P-O antysym stretching	[25,28,29,59,64,65,67,68,69,70]
υ_3_ PO_4_^3−^ HAp	1038–1040	P-O antysym stretching	[25,28,29,59,64,65,67,68,69,70]
υ_3_ PO_4_^3−^ HAp	1042	P-O antysym stretching	[25,28,29,59,64,65,67,68,69,70]
υ_3_ PO_4_^3−^ HAp	1047	P-O antysym stretching	[25,28,29,59,64,65,67,68,69,70]
υ_3_ PO_4_^3−^	1051–1052	P-O antysym stretching	[25,28,29,59,64,65,67,68,69,70]
υ_3_ PO_4_^3−^ß-TCP/HAp	1055	P-O antysym stretching	[57,67,68,77]
υ_3_ PO_4_^3−^ß-TCP/HAp	1062–1063	P-O antysym stretching	[25,28,29,59,64,65,67,68,69,70]
υ_1_ СO_3_ B-type	1069–1070	PO_4_ by CO_3_ substitution	[25,28,29,59,64,65,67,68,69,70]
υ_3_ PO_4_^3−^	1075–1076	P-O antysym stretching	[25,28,29,59,64,65,67,68,69,70]
υ_3_ PO_4_^3−^ß-TCP	1081–1082	P-O antysym stretching	[19,67,68,71,72,73]
υ_3_ PO_4_^3−^DCPD	1087–1088	P-O antysym stretching	[67,77]
CH_2_	2870–2880	Amino acid buster	[78,79]
CH3	2915–2935	Amino acid buster	[78,79]
CH	2960–2970	Amino acid buster	[78,79]
hydroxyl OH group	3570	OH stretching	[25,28,29,59,64,65,67,68,69,70]

**Table 4:** Carbonation levels for the apatite samples.

Sample	Raman Line Positions, cm^−1^		υ_1_ PO_4_^3−^ Peak FWHM, cm^−1^	Wt% (CO_3_)
	υ_1_ PO_4_^3−^	υ_1_ CO_3_ B-type		
CHAp-1	961.6	1069.3	9.97	1.85
CHAp-2	961.7	1069.3	9.87	1.80
CHAp-3	961.1	1069.5	9.70	1.71
BHN-1	961.6	1069.5	11.1	1.84
BHN-2	961.2	1069.7	10.7	1.75

## Data Availability

The data that support the findings of this study are available from the corresponding author upon reasonable request.
